# Using machine learning to evaluate a non-selective enhanced recovery pathway for hip and knee arthroplasty: perioperative factors associated with same-day and early discharge

**DOI:** 10.1186/s42836-026-00435-1

**Published:** 2026-10-12

**Authors:** Amr Selim, Abdelrahman Ibrahim, Abdul-Rahman Gomaa, Niall Graham, Geraint Thomas

**Affiliations:** 1 Robert Jones & Agnes Hunt Orthopaedic Hospital, Oswestry, UK; 2 School of Medicine, Keele University, Staffordshire, UK; 3 University Hospital of North Midlands, Stoke-On-Trent, UK

**Keywords:** Machine Learning, Enhanced Recovery after Surgery, Same-Day Discharge, Length of Stay, Total Hip Arthroplasty, Total Knee Arthroplasty, Unicompartmental Knee Arthroplasty, Spinal Anaesthesia, SHAP, Perioperative Care

## Abstract

**Introduction:**

Same-day and early discharge after total hip and knee arthroplasty (THA/TKA) depend not only on patient characteristics but on the perioperative pathway itself. This study used supervised machine learning (ML) to evaluate a universal, non-selective enhanced recovery after surgery (ERAS) pathway at a high-volume tertiary centre, identifying the perioperative factors most strongly associated with failure of Day 0 or Day 1 discharge, and of same-day (Day 0) discharge.

**Methods:**

All patients undergoing primary THA, TKA, or partial knee replacement (PKR) within a universal, non-selective ERAS pathway (April 2024–May 2025) were included, with no exclusion on the basis of age, comorbidity, or anticipated day-case suitability. ML models (Logistic Regression, Random Forest, Gradient Boosting, Support Vector Machine, Decision Tree) were developed in Python using the full set of preoperative, anaesthetic, and postoperative pathway variables, with SHAP used for interpretation.

**Results:**

A total of 3,025 patients were included (median age 70.1 years, IQR 62.3–76.6). With discharge failure as the positive class, test ROC-AUC for failure of Day 0 or Day 1 discharge ranged from 0.66 to 0.76 (PR-AUC 0.34–0.50; baseline failure prevalence 18%). For same-day discharge failure, ROC-AUC ranged from 0.76 to 0.82 (F1 0.76–0.83; PR-AUC 0.89–0.93; baseline prevalence 73%). SHAP analysis ranked the factors most strongly associated with discharge failure. For same-day discharge, time to mobilisation was the most significant factor, followed by postoperative radiography before return to the ward and mobiliser designation. For Day 0 or Day 1 discharge, the strongest associations were ASA grade, sex, operative side, and time to mobilisation.

**Conclusion:**

Supervised ML is an effective framework for evaluating a non-selective ERAS pathway. Time to mobilisation dominated same-day discharge and was a leading factor for Day 0 or Day 1 discharge; it warrants prospective confirmation as a modifiable target.

**Supplementary Information:**

The online version contains supplementary material available at https://doi.org/10.1186/s42836-026-00435-1.

## Introduction

Day Case and Enhanced Recovery After Surgery (ERAS) protocols for hip and knee arthroplasty have been widely adopted to accelerate recovery, improve outcomes, shorten hospital stays, reduce costs, and standardize care [1–5]. In parallel, increasing demand for elective arthroplasty, workforce constraints, and pressure on inpatient bed capacity have intensified the need for reliable and efficient early discharge pathways.

ERAS, unlike day-case surgery, does not require pre-selection of healthy or low-risk patients [1, 6, 7]. Instead, discharge depends on meeting criteria such as adequate pain control, mobility and clinical stability [7]. Identifying perioperative factors associated with failure to achieve Day 0 or Day 1 discharge is therefore essential for perioperative planning, resource allocation and optimisation of modifiable risk factors [8].

This study uses supervised machine learning (ML) to evaluate a universal, standardised, non-selective ERAS pathway implemented at a high-volume tertiary orthopaedic centre, and to identify which components of that pathway are most strongly associated with failure of Day 0 or Day 1 discharge. Because preoperative characteristics alone only partly explain discharge outcomes and have been studied previously, we did not aim to predict discharge from preoperative factors. Instead, we evaluated the whole pathway—including anaesthetic, analgesic and rehabilitation components—to identify potentially modifiable steps for prospective evaluation. ML algorithms with feature importance analysis are well suited to this task, as they accommodate the full set of perioperative variables simultaneously and can reveal complex, non-linear interactions between them [9]. When combined with interpretable techniques such as SHAP (SHapley Additive exPlanations) analysis, these models may facilitate translation into routine clinical practice and service-level decision-making.

## Methods

All patients undergoing primary total hip arthroplasty (THA), total knee arthroplasty (TKA), or partial knee replacement (PKR) under a universal non-selective ERAS pathway between April 2024 and May 2025 were included.

**Fig. 1:**
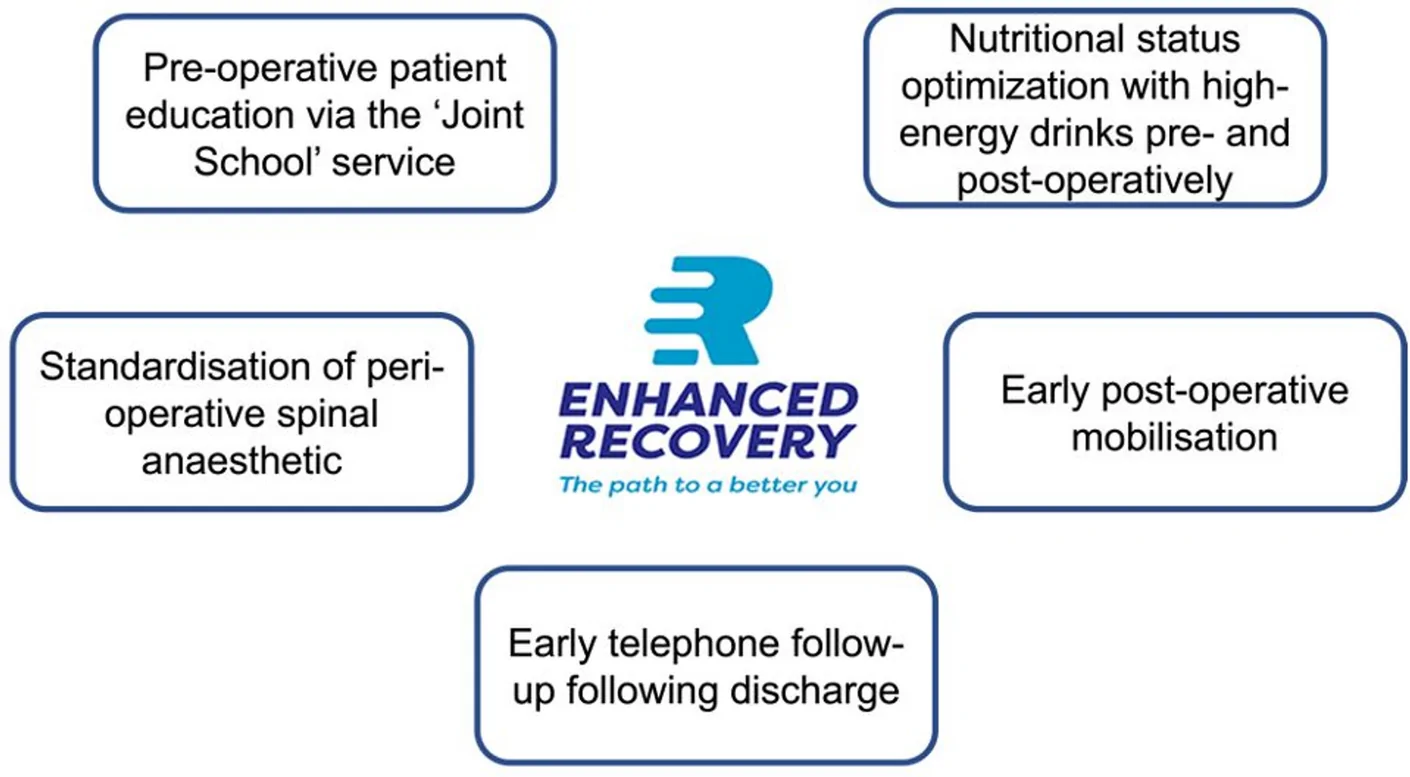
Components of the ERAS Pathway

**Fig. 2:**
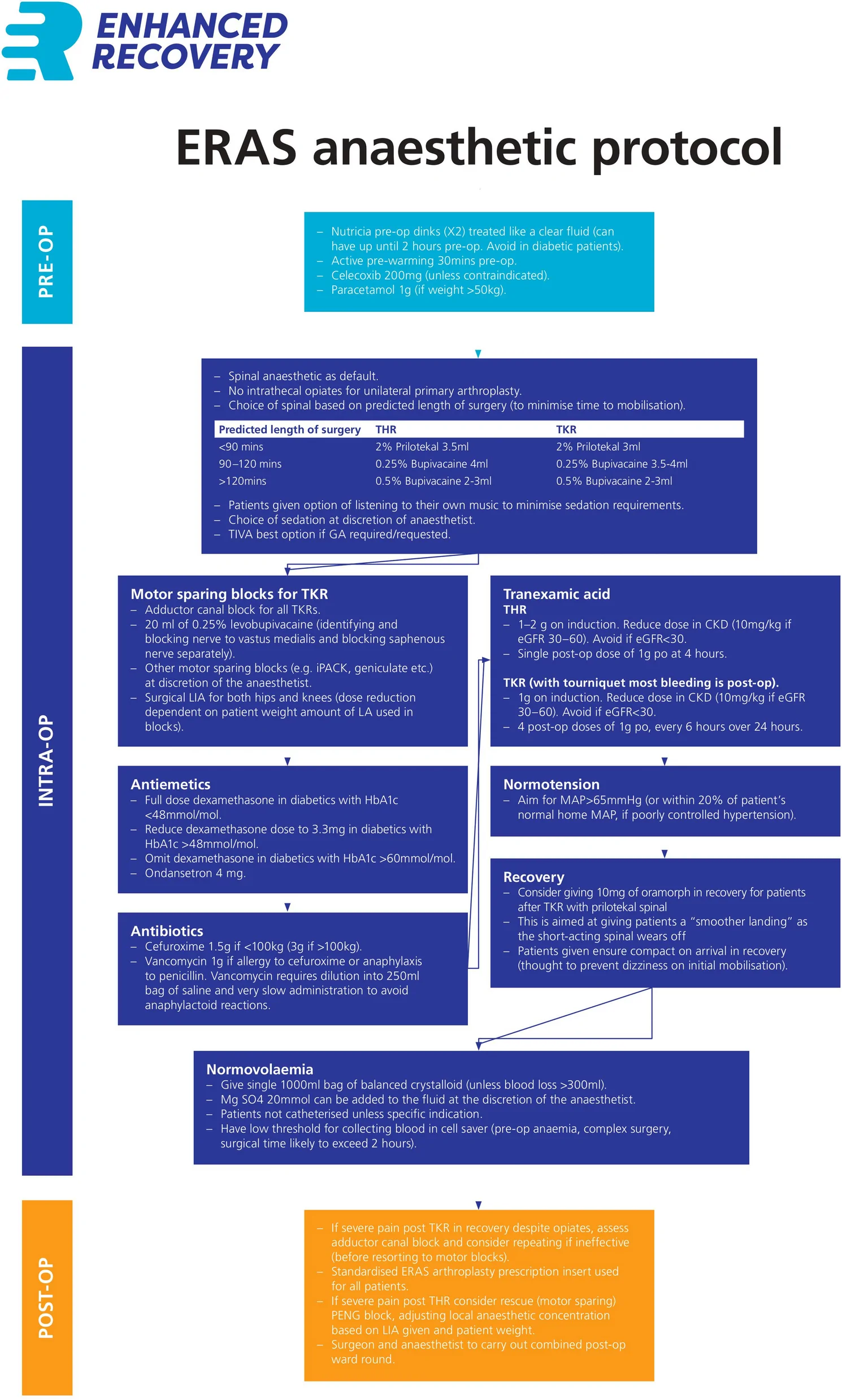
Anaesthetic Protocol for the ERAS Pathway

No patients were excluded because of age, comorbidity, or anticipated suitability for day-case surgery, which is why the pathway is described as non-selective. The cohort therefore spanned the full range of surgical risk, including 540 patients (19.0%) of ASA grade 3 or 4, 410 (13.6%) aged 80 or older, and 33 bilateral procedures. The study evaluated an established pathway rather than developing a preoperative prediction tool. Time to mobilisation was measured from return to the ward or high-dependency unit to first mobilisation, both recorded prospectively. The derived value agreed with these two source clock times to within 15 min in 98.1% of records in which both were available (2,865/2,921), supporting its use as an auditable derived variable. Mobilisation was defined as getting out of bed and taking the first steps. The pathway comprises preoperative education, nutritional support, a standardised anaesthetic and analgesic regimen, early physiotherapy and structured telephone follow-up (Fig. 1). The detailed anaesthetic protocol is outlined in Fig. 2.

Prospectively collected clinical data were analysed. The study was exempt from Institutional Review Board approval, as all data were routinely collected for clinical care and processed anonymously. Missing data were managed using a predefined, variable-specific imputation strategy. For true missing values, continuous variables were imputed using Multivariate Imputation by Chained Equations (MICE), while categorical variables were imputed using the most frequent category. All imputation was fitted within the training partition and applied to held-out data. Structurally non-applicable values were not treated as missing. Spinal volume in patients without a spinal, dexamethasone dose in those not given it, and nutritional volumes in those not supplemented were coded as zero to reflect the quantity administered. Spinal drug type and preparation were instead given a separate “not given” category, since zero already denotes bupivacaine and plain preparation. Missingness was low, highest for ASA grade at 5.95% (*n* = 180) and below 3.1% for all other predictors. The extent of missingness and the imputation method applied to each variable are summarised in Supplementary Table A.

**Fig. 3:**
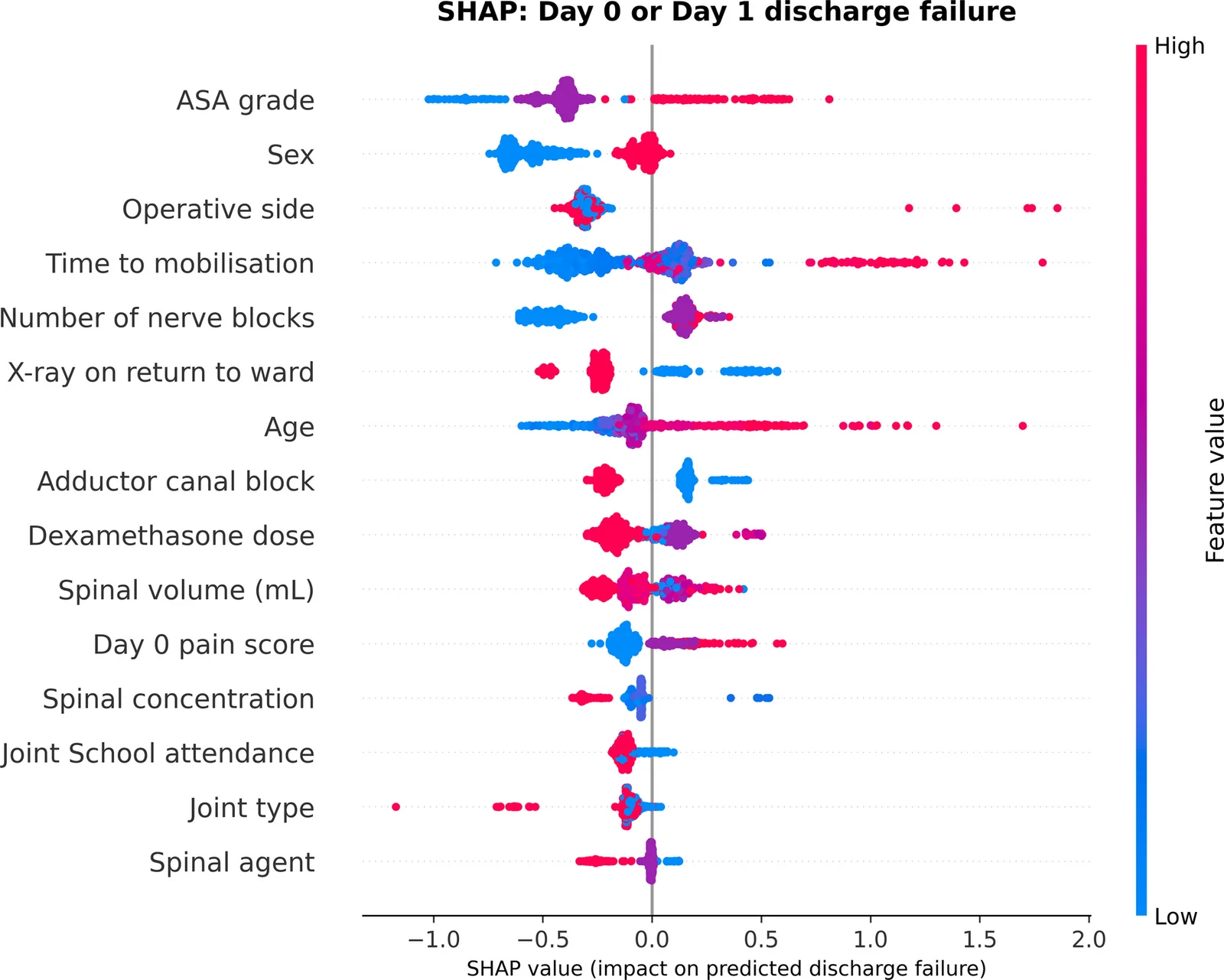
SHAP feature importance for Day 0 or Day 1 discharge failure, computed from the Gradient Boosting model on the held-out test partition (*n* = 605). Features are ordered by mean absolute SHAP value (Supplementary Table F); positive values indicate contributions towards prediction of the positive class, discharge failure. SHAP rankings are model-dependent. A cross-model comparison is provided in Supplementary Table J

**Fig. 4:**
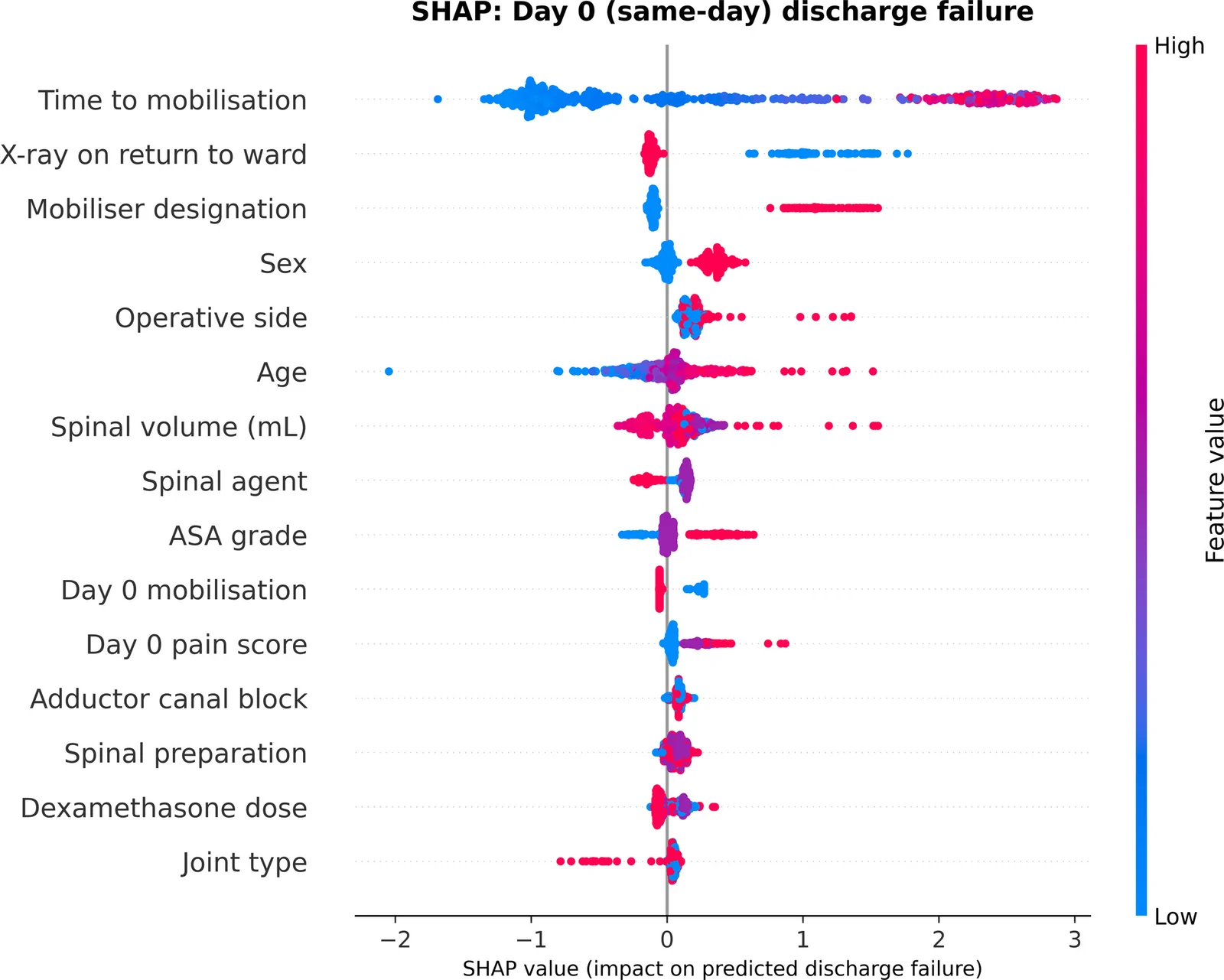
SHAP feature importance for Day 0 (same-day) discharge failure, computed from the Gradient Boosting model on the held-out test partition (*n* = 605). Features are ordered by mean absolute SHAP value (Supplementary Table G); positive values indicate contributions towards prediction of the positive class, discharge failure. SHAP rankings are model-dependent; a cross-model comparison is provided in Supplementary Table J

Five supervised ML models were developed: logistic regression, random forest, gradient boosting, support vector machine (SVM), and decision tree. Two outcomes were modelled as markers of pathway performance: failure to discharge at Day 0 or Day 1, and failure of same-day (Day 0) discharge. Internal validation used an 80/20 stratified train-test split. All primary results (Tables 4 and 5, Figs. 3 and 4) were derived from this split; the chronological 2024/2025 split described below provided a separate temporal validation (Supplementary Tables C and D). To address class imbalance, SMOTE (Synthetic Minority Over-sampling Technique) was applied within the training data only. Hyperparameters were predefined from prior optimisation and clinical interpretability rather than grid searching. Values are reported in Supplementary Table I, with scikit-learn defaults elsewhere. The train-test split, SMOTE, and all models used random_state = 42, with a classification threshold of 0.5. Imputation, SMOTE resampling, and classifier fitting were confined to the training partition, and SHAP values were computed only on the held-out test partition. Analyses were conducted in Python 3.12.3 using scikit-learn 1.8.0, imbalanced-learn 0.14.2, and SHAP 0.52.0. Continuous variables were assessed for skewness. Approximately symmetric variables are reported as mean (SD) and compared using t-tests, and skewed variables as median (IQR) and compared using Mann–Whitney U tests. Categorical variables were compared using chi-square tests without continuity correction, or Fisher’s exact test where any cell contained fewer than ten observations. P-values are reported to three decimal places.

Temporal validation used a chronological split, with patients treated in 2024 for model training (*n* = 1,898) and those treated in 2025 reserved as a temporally independent test set (*n* = 1,127), in accordance with TRIPOD-AI recommendations. The completed TRIPOD-AI checklist is provided as Supplementary Material N. Performance was evaluated using ROC-AUC, PR-AUC, precision, recall, F1 score, Brier score, and confusion matrices.

Variables comprised patient demographics, ASA grade, laterality and joint type; Joint School attendance and pre-operative nutrition; anaesthetic technique, spinal drug type, preparation, concentration and volume, dexamethasone dose and nerve block use; and postoperative radiography, pain severity, blood transfusion, time to first mobilisation, nutritional supplementation and mobiliser designation. Spinal agent and volume were modelled separately; the derived variable spinal dose was not used as a predictor, because bupivacaine and prilocaine doses do not overlap (2.5–33 vs. 34–110 mg) and dose would act as a proxy for agent. The full variable list is given in Supplementary Table A.

Global feature importance was summarised using mean absolute SHAP values, with the top 15 predictors visualised for each outcome.

All code used for preprocessing, model development, validation and SHAP generation is provided in Supplementary Material B.

## Results

A total of 3,025 patients were included (median age 70.1 years, IQR 62.3–76.6; 49.8% female; 73.5% ASA grade 2), comprising 44.4% THA, 51.5% TKA and 4.1% PKR (Tables 1, 2 and 3). Median length of stay was 1 day (IQR 0–1) overall and 0 days for PKR (*p* < 0.001), with a median time to discharge of 29 h (IQR 12.5–33). Same-day (Day 0) discharge occurred in 811 patients (26.8%) and Day 0 or Day 1 discharge in 2,481 (82.0%).

**Table 1:** Patient demographics and preoperative variables

Variable	Category	Statistic
Age	Median (IQR)	70.1 (62.3–76.6) years
Sex	Male	1,519 (50.21%)
	Female	1,506 (49.79%)
ASA Status †	1	213 (7.49%)
	2	2,092 (73.53%)
	3	537 (18.88%)
	4	3 (0.11%)
	Missing	180 (5.95%)
Joint Type †	THA	1,343 (44.41%)
	TKA	1,558 (51.52%)
	PKR	123 (4.07%)
	Missing	1 (0.03%)
Side	Right	1,590 (52.56%)
	Left	1,402 (46.35%)
	Bilateral	33 (1.09%)
Attended Joint School	Yes	2,567 (84.86%)
	No	458 (15.14%)
Pre-op Nutrition Given	Yes	2,340 (77.36%)
	No	685 (22.64%)
Pre-op Nutrition Volume ‡	Median (IQR)	400 (400–400) mL

*N* = 3,025 unless otherwise stated

^†^ Percentages calculated on non-missing denominators (ASA *n* = 2,845; joint type *n* = 3,024); missing counts are shown separately and expressed as a percentage of the total cohort

^‡^ Among patients receiving pre-operative nutrition (*n* = 2,340; 2 missing volumes). Age is reported as median (IQR) as the distribution was skewed (skewness − 0.74)

THA = total hip arthroplasty; TKA = total knee arthroplasty; PKR = partial knee replacement, comprising 117 unicompartmental and 6 patellofemoral replacements

**Table 2:** Anaesthetic variables

Variable	Category	Statistic
Spinal Anaesthetic Given §	Yes	2,824 (93.36%)
	No	201 (6.64%)
Spinal Only §	Yes	2,698 (89.19%)
	No	327 (10.81%)
General Anaesthesia §	Yes	311 (10.28%)
	No	2,714 (89.72%)
Anaesthetic Technique §	Spinal only	2,698 (89.19%)
	Spinal + GA	126 (4.17%)
	GA only	185 (6.12%)
	Neither recorded	16 (0.53%)
Spinal Volume ¶ ‖	Mean (SD)	3.29 (0.68) mL
Spinal Type ¶	Bupivacaine	2,194 (77.69%)
	Prilocaine	626 (22.17%)
	Not recorded	4 (0.14%)
Spinal Concentration ¶	Bupivacaine 0.25%	921 (32.61%)
	Bupivacaine 0.375%	36 (1.27%)
	Bupivacaine 0.5%	1,237 (43.80%)
	Prilocaine 2%	626 (22.17%)
Spinal Preparation ¶ #	Bupivacaine – Plain	1,563 (71.24% of bupivacaine)
	Bupivacaine – Heavy	631 (28.76% of bupivacaine)
	Prilocaine – Heavy	626 (100% of prilocaine)
	Not recorded	4 (0.14%)
Bupivacaine Dose **	Median (IQR)	11.0 (10.0–15.0) mg
Prilocaine Dose **	Median (IQR)	70.0 (64.0–70.0) mg
Dexamethasone Given §	Yes	2,788 (92.17%)
	No	237 (7.83%)
Dexamethasone Dose ††	Median (IQR)	13.2 (6.6–13.2) mg
Nerve Block Given ‡‡	Yes	1,682 (55.95%)
	No	1,324 (44.05%)
	Missing	19 (0.63%)
Number of Nerve Blocks §	0	1,360 (44.96%)
	1	1,466 (48.46%)
	2	195 (6.45%)
	3	4 (0.13%)
Anaesthetic Protocol Followed ‡‡	Yes	2,719 (91.77%)
	No	244 (8.23%)
	Missing	62 (2.05%)

^§^ Percentage of the total cohort (*N* = 3,025)

^¶^ Percentage of spinal recipients (*n* = 2,824)

^‡‡^ Percentage of non-missing values; missing counts are expressed as a percentage of the total cohort

‖ Spinal volume is reported as mean (SD) as the distribution was approximately symmetric (skewness − 0.01); *n* = 2,756 (68 missing)

^#^ All prilocaine was administered as a 2% hyperbaric (heavy) preparation; the plain/heavy distinction is therefore informative only within bupivacaine administrations, and baricity comparisons are reported within bupivacaine only

^**^ Doses calculated as concentration (%) × 10 mg/mL × volume (mL) and reported as median (IQR) as the distributions were skewed

^††^ Among patients receiving dexamethasone (*n* = 2,788; 4 missing doses). Anaesthetic technique categories are mutually exclusive

**Table 3:** Postoperative and outcome variables

Variable	Category	Statistic
Post-op Nutrition Given	Yes	1,520 (50.25%)
	No	1,505 (49.75%)
Post-op Nutrition Volume §§	125 mL	768 (50.53%)
	250 mL	752 (49.47%)
X-ray on Return to Ward	Yes	2,450 (80.99%)
	No	575 (19.01%)
Day 0 Pain ‡‡	None	2,168 (72.39%)
	Mild	624 (20.83%)
	Moderate	172 (5.74%)
	Severe	31 (1.04%)
	Missing	30 (0.99%)
Day 0 Mobilisation ‡‡	Yes	2,276 (77.05%)
	No	678 (22.95%)
	Missing	71 (2.35%)
First Mobiliser ‡‡	Physiotherapist/ER Practitioner	2,498 (85.02%)
	Nurse/HCA	440 (14.98%)
	Missing	87 (2.88%)
Time to Mobilisation ¶¶	Median (IQR)	2.9 (1.8–6.0) hours
Blood Transfusion ‡‡	Yes	24 (0.82%)
	No	2,910 (99.18%)
	Missing	91 (3.01%)
Day 0 (Same-Day) Discharge	Yes	811 (26.81%)
	No	2,214 (73.19%)
Discharge Day 0 or Day 1	Yes	2,481 (82.02%)
	No	544 (17.98%)
Time to Discharge ¶¶	Median (IQR)	29.4 (12.5–33.0) hours
Length of Stay ¶¶	Median (IQR)	1 (0–1) day

^‡‡^ Percentage of non-missing values; missing counts are expressed as a percentage of the total cohort (*N* = 3,025)

^§§^ Among patients receiving post-operative nutrition (*n* = 1,520); only two volumes were administered, so counts are reported rather than a summary statistic

^¶¶^ Reported as median (IQR) as the distributions were skewed (time to mobilisation skewness + 2.99; time to discharge + 20.1; length of stay + 19.9)

### Predictors of Day 0 or Day 1 discharge

Day 0 or Day 1 discharge was more likely in younger patients (median 69.4 [IQR 61.8–75.9] vs. 73.5 [65.2–80.2] years; Mann–Whitney U), men (85.6% vs. 78.4%), lower ASA grade (93.0%, 86.4% and 62.9% for grades 1–3), unilateral rather than bilateral procedures (82.6% vs. 30.3%) and PKR (95.1%, vs. THA 82.0% and TKA 81.0%); all *p* < 0.001. Joint School attendance (82.8% vs. 77.7%; *p* = 0.010) and pre-operative nutrition (83.5% vs. 76.8%; *p* < 0.001) were also associated with earlier discharge. Discharge rates varied by anaesthetic technique, being highest with spinal anaesthesia alone (83.2%; 2,246/2,698), intermediate with combined spinal and general anaesthesia (74.6%; 94/126), and lowest with general anaesthesia alone (70.8%; 131/185), with a significant trend across increasing exposure to general anaesthesia (*p* < 0.001 for both the overall comparison and the trend). Prilocaine was associated with a higher rate than bupivacaine (89.8% vs. 80.9%; *p* < 0.001), as was lower bupivacaine concentration (0.25%:83.3%; 0.5%:79.1%; *p* = 0.045). Within bupivacaine, higher spinal volumes were marginally associated with earlier discharge (mean 3.26 vs. 3.16 mL; *p* = 0.021) but not within prilocaine (*p* = 0.066), and heavy preparations were associated with a lower rate than plain (77.7% vs. 82.2%; *p* = 0.014). Dexamethasone (82.5% vs. 76.4%; *p* = 0.018) and protocol adherence (83.2% vs. 67.2%; *p* < 0.001) were also associated with earlier discharge. Pathway factors showed the steepest gradients. Discharge was more likely in patients who had radiography on return to the ward than in those who did not (86.3% vs. 63.7%), in those mobilised on the day of surgery (86.0% vs. 69.8%), in those given post-operative nutritional supplementation (87.6% vs. 76.4%; all *p* < 0.001), and when first mobilisation was led by a physiotherapist or enhanced-recovery practitioner rather than nursing staff (83.0% vs. 78.0%; *p* = 0.010). Patients discharged by Day 1 had mobilised sooner than those who were not (median 2.7 vs. 4.7 h; *p* < 0.001). Discharge became progressively less likely as Day 0 pain increased (83.9%, 79.8%, 74.4% and 71.0% for no, mild, moderate and severe pain; *p* < 0.001), and was markedly less likely in patients requiring blood transfusion, a marker of perioperative blood loss [10, 11] (33.3% vs. 83.6%; *p* < 0.001). Overall, nerve block use was not associated with Day 0 or Day 1 discharge (block 81.5% vs. no block 82.7%; *p* = 0.397), but block type was informative within knee replacement. Compared with knees that did not receive them, discharge was more likely after an adductor canal block (82.3% vs. 65.9%) and less likely after fascia iliaca blockade (60.5% vs. 82.1%); both *p* < 0.001. In hip replacement, PENG blockade was not associated with discharge (74.5% vs. 82.3%; *p* = 0.143). More blocks were associated with lower discharge rates in both hip (Spearman ρ =  − 0.077; *p* = 0.005) and knee replacement (ρ =  − 0.050; *p* = 0.049).

### Predictors of same-day (Day 0) discharge

The same factors were associated with same-day discharge. Patients discharged the same day were younger (median 68.0 [IQR 60.7–74.6] vs. 70.8 [63.1–77.1] years), more often men (31.1% vs. 22.5%), and of lower ASA grade (35.2%, 29.3% and 15.5% for grades 1–3); all *p* < 0.001, including the ASA trend. It also varied by joint type (*p* < 0.001), an effect driven entirely by PKR (54.5%), with no difference between THA and TKA (26.0% vs. 25.3%; *p* = 0.668). Joint School attendance remained associated with same-day discharge (attended 27.5% vs. 22.9% in non-attenders; *p* = 0.042), whereas pre-operative nutritional supplementation (27.4% vs. 24.7%; *p* = 0.151) and laterality (unilateral 27.0% vs. bilateral 12.1%; *p* = 0.073, Fisher’s exact) were not. Discharge rates followed the same ordering by anaesthetic technique: 27.7% (747/2,698) with spinal anaesthesia alone, 22.2% (28/126) with combined spinal and general anaesthesia, and 17.8% (33/185) with general anaesthesia alone (*p* = 0.007 overall; *p* = 0.002 for trend). Discharge was more likely after prilocaine than after bupivacaine (42.8% vs. 23.0%; *p* < 0.001). Among bupivacaine administrations, it also varied by concentration (*p* = 0.004), being highest at 0.25% (26.2%) and lower at 0.5% (21.0%) and 0.375% (11.1%, *n* = 36). Higher spinal volumes were marginally associated with same-day discharge within bupivacaine (mean 3.30 vs. 3.23 mL; *p* = 0.047) but not within prilocaine (*p* = 0.194), and baricity was not associated with it (heavy 20.9% vs. plain 23.9%; *p* = 0.138). Dexamethasone administration (27.3% vs. 21.1%; *p* = 0.039) and adherence to the anaesthetic protocol (27.8% vs. 16.8%; *p* < 0.001) were both associated with same-day discharge. Pathway factors again showed by far the largest differences. Same-day discharge was more likely in patients who had radiography on return to the ward (32.4% vs. 3.0%), whose first mobilisation was led by a physiotherapist or enhanced-recovery practitioner rather than nursing staff (31.9% vs. 2.5%), and who received post-operative nutritional supplementation (32.6% vs. 20.9%); all *p* < 0.001. Patients discharged the same day had also mobilised sooner than those who were not (median 1.8 vs. 3.7 h; *p* < 0.001). Discharge was less likely in the 24 patients who required blood transfusion than in those who did not (8.3% vs. 27.5%; *p* = 0.037, Fisher’s exact), whereas Day 0 pain severity was not associated with same-day discharge (27.6%, 27.1%, 20.3% and 22.6% for no, mild, moderate and severe pain; *p* = 0.209). Nerve block use overall was not associated with same-day discharge (27.2% vs. 26.2%; *p* = 0.530), nor was PENG blockade in hip replacement (20.0% vs. 26.2%; *p* = 0.301). Within knee replacement the adductor canal block was again associated with a higher rate (26.0% vs. 17.1%; *p* = 0.029) and a shorter median time to mobilisation (2.7 vs. 3.7 h), and fascia iliaca blockade with a lower rate (13.6% vs. 25.9%; *p* = 0.013) and a longer time to mobilisation (5.5 vs. 2.7 h; *p* < 0.001) despite less moderate or severe pain (4.9% vs. 7.0%). The number of blocks showed no association with same-day discharge in either joint (hip ρ =  − 0.035, *p* = 0.201; knee ρ =  − 0.016, *p* = 0.536). Odds ratios with 95% confidence intervals for all univariate comparisons, for both outcomes, are given in Supplementary Table M.

Discharge rates fell steadily as time to first mobilisation increased. Same-day discharge fell from 48.2% among patients mobilised within two hours to 32.2%, 16.5% and 1.3% at two to four, four to six and six to twelve hours, and none beyond twelve hours (Spearman ρ =  − 0.414; *p* < 0.001). Discharge by Day 1 followed the same order but far less steeply, from 91.1% to 85.0%, 78.1%, 74.5% and 68.6% (ρ =  − 0.235; *p* < 0.001).

Models were refitted separately for hip and knee replacement. Logistic regression was used throughout, so that subgroup and pooled models could be compared directly.

Hip-specific models performed better than the pooled model, with ROC-AUC rising from 0.809 to 0.883 for same-day discharge failure and from 0.760 to 0.833 for Day 0 or Day 1 failure. Knee-specific models showed no such gain (0.814 and 0.730 against 0.809 and 0.760). Partial knee replacement was not modelled separately: with 123 patients and only six Day 0 or Day 1 failures, there were 0.2 events per candidate predictor, far below the number needed for stable estimation. Time to mobilisation ranked first in both joint-specific models (mean absolute SHAP 1.20 for hip, 1.14 for knee), whereas radiography on return to the ward ranked second for hip but fifth for knee (0.53 vs. 0.22). Full results are given in Supplementary Table L.

### Machine learning models

Discharge failure was the positive class for both outcomes. For failure of Day 0 or Day 1 discharge, discrimination was moderate, with ROC-AUC between 0.66 and 0.76 and PR-AUC between 0.34 and 0.50 against a baseline failure prevalence of 18%. Classification performance was correspondingly limited, with precision from 0.34 to 0.53, recall from 0.28 to 0.66, and F1 from 0.36 to 0.45 (Table 4). Logistic regression achieved both the highest ROC-AUC and the highest F1 for this outcome. For failure of same-day discharge, performance was higher and more stable, with ROC-AUC between 0.76 and 0.82 and PR-AUC between 0.89 and 0.93 against a baseline failure prevalence of 73%. Precision ranged from 0.82 to 0.91, recall from 0.66 to 0.82, and F1 from 0.76 to 0.83 (Table 5). Because failure was the majority class for this outcome, the high F1 and PR-AUC values partly reflect that prevalence and should be read against it. Gradient boosting achieved both the highest ROC-AUC and the highest F1 for this outcome. Logistic regression and the support vector machine showed the smallest training-to-test difference. On temporally independent data, the decision tree declined most (0.663 to 0.611 for Day 0 or Day 1 discharge), while every other model changed by 0.017 or less (Supplementary Tables C and D; calibration curves and confusion matrices in Supplementary Material E and Supplementary Fig. E).

**Table 4:** Model performance—failure of Day 0 or Day 1 discharge (80/20 stratified split)

Model	ROC-AUC	PR-AUC	Precision	Recall	F1	Brier
Logistic Regression	0.760	0.497	0.344	0.661	0.453	0.200
Support Vector Machine	0.757	0.493	0.341	0.651	0.448	0.200
Random Forest	0.728	0.390	0.517	0.275	0.359	0.137
Gradient Boosting	0.744	0.436	0.530	0.321	0.400	0.132
Decision Tree	0.663	0.336	0.459	0.358	0.402	0.163

Positive class = failure of Day 0 or Day 1 discharge. Baseline prevalence of the positive class = 18.0%; a no-skill classifier would therefore achieve a PR-AUC of approximately 0.18 for this outcome. Test set *n* = 605. Imputation was fitted within the training partition only, and SMOTE was applied to the training data only. PR-AUC = precision–recall area under the curve

**Table 5:** Model performance—failure of Day 0 (same-day) discharge (80/20 stratified split)

Model	ROC-AUC	PR-AUC	Precision	Recall	F1	Brier
Logistic Regression	0.809	0.930	0.910	0.664	0.768	0.181
Support Vector Machine	0.806	0.929	0.906	0.655	0.760	0.181
Random Forest	0.811	0.931	0.840	0.817	0.828	0.155
Gradient Boosting	0.815	0.931	0.837	0.822	0.829	0.153
Decision Tree	0.761	0.886	0.818	0.792	0.805	0.192

Positive class = failure of Day 0 discharge. Baseline prevalence of the positive class = 73.2%; a no-skill classifier would therefore achieve a PR-AUC of approximately 0.73 for this outcome. The high F1 and PR-AUC values consequently reflect the majority-class prevalence in part and should be interpreted against this baseline. Test set *n* = 605. Imputation was fitted within the training partition only, and SMOTE was applied to the training data only

SHAP values were computed on the held-out test partition. For failure of Day 0 or Day 1 discharge (Fig. 3), the highest-ranked features were ASA grade, sex, operative side, time to mobilisation, number of nerve blocks, radiography on return to the ward, age and adductor canal block (Supplementary Table F). For failure of same-day discharge (Fig. 4), time to mobilisation was clearly dominant, followed by radiography on return to the ward, designation of the first mobilising practitioner, sex, operative side and age (Supplementary Table G). Prilocaine rather than bupivacaine, higher spinal volumes and higher dexamethasone doses each pushed predictions towards discharge rather than failure, whereas hip or knee rather than partial knee replacement pushed towards failure.

## Discussion

Identifying which components of a non-selective ERAS pathway are most strongly associated with failure of early discharge is essential for refining perioperative care and allocating resources. Whereas earlier work examined preoperative predictors of length of stay, this study evaluated the pathway as a whole, weighing preoperative, anaesthetic and postoperative components within a single framework. Time to first mobilisation was the strongest single correlate of discharge. It ranked first in the same-day discharge model with a mean absolute SHAP value four times that of the next variable, first in all three model types, and first in both joint subgroups, and discharge rates fell steadily across every band of mobilisation timing. This is consistent with evidence that day-of-surgery mobilisation shortens stay [12, 13]. For Day 0 or Day 1 discharge, two fixed patient characteristics ranked highest—ASA grade first and sex second—with operative side third and time to mobilisation fourth.

Performance differed by algorithm. Logistic regression and the SVM generalised best, whereas the tree-based models showed much larger falls in ROC-AUC from training to test data (random forest 0.13–0.25; decision tree 0.16–0.25; logistic regression 0.02–0.04), consistent with overfitting at the specified tree depth. The high PR-AUC for same-day discharge failure reflects both model performance and the high baseline prevalence of the positive class (73%), and should be interpreted against that baseline rather than as evidence of superior discrimination. The further decline to temporally independent validation was modest for every model. We therefore asked whether practice itself had changed between 2024 and 2025. Case mix and discharge outcomes were stable (same-day discharge 27.1% vs. 26.3%, *p* = 0.602; Day 0 or Day 1 82.2% vs. 81.7%, *p* = 0.745), but four process measures shifted towards greater compliance: anaesthetic protocol adherence, Joint School attendance, spinal-only anaesthesia and mean spinal volume (all *p* ≤ 0.042; Supplementary Table K). The pathway therefore matured while its outputs remained stable. Because practice shifted within a single year, any deployed model would need periodic refitting, with logistic regression or the SVM preferable to the tree-based models.

Older age, female sex, and higher ASA grade were consistently associated with discharge failure, supporting pre-admission planning and targeted allocation of physiotherapy and nursing resources [7, 8]. Procedure type mattered only for partial knee replacement; hip and knee replacement did not differ (*p* = 0.499 and 0.668). Several factors showed significant univariate associations but low SHAP importance, because the two measures answer different questions: univariate testing asks whether an association exists, and SHAP asks how much a variable contributes to prediction. Joint School attendance illustrates this. It was associated with earlier discharge univariately (82.8% vs. 77.7%; *p* = 0.010) but contributed little to prediction, reflecting near-universal attendance (84.9%), a modest effect size (unadjusted OR 1.34, 95% CI 1.04–1.73), and strong co-occurrence with pre-operative nutrition (79.4% vs. 65.9%; *p* < 0.001). After adjustment, the association with Day 0 or Day 1 discharge was no longer significant (OR 1.23, 0.94–1.63), whereas that with same-day discharge persisted (OR 1.38, 1.07–1.77; *p* = 0.013). Core education at routine clinic visits may therefore suffice in resource-constrained settings [7, 14, 15].

Several anaesthetic factors were also associated with discharge. Spinal anaesthesia alone was associated with earlier discharge for both outcomes, in keeping with literature linking general anaesthesia to nausea, vomiting, delayed mobilisation and pain [16, 17]. Within bupivacaine administrations, heavy preparations were associated with a lower rate of Day 0 or Day 1 discharge than plain preparations, with no difference for same-day discharge. Baricity was therefore not identified as a factor favouring earlier discharge in this cohort [18]. Dexamethasone was associated with earlier discharge, although optimal dosing remains debated [19, 20].

Peripheral nerve block use overall was not associated with either outcome, but block type was informative within knee replacement [21]. The motor-sparing adductor canal block specified by the pathway was associated with higher discharge rates for both outcomes, alongside a shorter time to first mobilisation. Fascia iliaca blockade showed the opposite pattern, with a longer time to first mobilisation despite comparable Day 0 analgesia. This dissociation between pain control and mobility is consistent with the quadriceps weakness that fascia iliaca blockade can produce[22], but the adductor canal block is designed to avoid this [23]. PENG blockade was not associated with either outcome in hip replacement. Since time to mobilisation was the dominant factor in the models, this suggests that anaesthetic and analgesic choices are best judged not by analgesia alone but by whether they preserve the ability to mobilise early.

Joint-specific modelling showed the dominant association was consistent across hip and knee replacement, with time to mobilisation ranking first in both (mean absolute SHAP 1.20 and 1.14). Secondary associations differed, with postoperative radiography more prominent in hip replacement. Hip-specific models also discriminated better than the pooled model, suggesting that pooling joint types obscures joint-specific structure.

Most ML work on arthroplasty discharge has addressed patient selection rather than pathway evaluation. Lopez et al. used neural networks to select candidates for outpatient arthroplasty (ROC-AUC 0.802 knee, 0.814 hip) [24], Turcotte et al. reported 0.773 from preoperative predictors alone [25], Langenberger applied ML to length of stay dichotomised at five days [8], and Feng et al. compared fourteen algorithms with SHAP interpretation in 449 ambulatory knee replacements (0.832) [26]. All used preoperative variables in patients pre-screened for day-case suitability to decide who enters a short-stay pathway. Two gaps follow. Performance in screened patients does not transfer to unselected practice: the studies above were developed in cohorts pre-selected for ambulatory suitability, whereas Gromov et al. achieved same-day discharge in only 15% of 557 all-comers [27]. And selection models cannot show which parts of a pathway are modifiable, because they omit the intraoperative and postoperative steps that constitute it. The present study addresses both, evaluating an unselected pathway in 3,025 consecutive patients and modelling anaesthetic, analgesic, nutritional, radiographic, mobilisation and staffing variables alongside patient characteristics. Its same-day rate of 26.8% sits well above the 15% reported in unselected practice, while remaining below the rates achieved with preoperative selection.

Four limitations should be noted. First, many top-ranked variables were recorded during the pathway and are unavailable preoperatively, limiting use for preoperative risk assessment. In sensitivity analysis using only preoperative and intraoperative variables, discrimination for same-day discharge fell from approximately 0.82 to 0.64, while Day 0 or Day 1 discharge changed less, from 0.76 to 0.72 (Supplementary Table H). Several key predictors may therefore reflect the discharge process rather than preceding causal factors. Second, SHAP rankings were model-dependent: time to mobilisation ranked first for same-day discharge across gradient boosting, random forest and logistic regression, but agreement was weaker for lower-ranked features (Supplementary Table J). Third, the routinely collected data did not record postoperative complications such as nausea and vomiting, orthostatic intolerance, urinary retention and haematoma. Day 0 pain, blood transfusion and time to mobilisation capture their effects only indirectly, so some associations may reflect unmeasured complications. Finally, this single-centre study requires external validation before wider application.

## Conclusion

Supervised machine learning is an effective framework for evaluating a non-selective ERAS pathway. Time to first mobilisation dominated same-day discharge and remained a leading factor for Day 0 or Day 1 discharge, where fixed patient characteristics ranked highest. Several are measured concurrently with discharge and require prospective confirmation before being treated as causal. This whole-pathway evaluation nonetheless indicates where the pathway can be strengthened.

## Supplementary Information

Supplementary Material 1.

Supplementary Material 2.

## Data Availability

The anonymised datasets used for the current study is available upon request by the editor or reviewers.
