# Supplementary material for: Using machine learning to evaluate a non-selective enhanced recovery pathway for hip and knee arthroplasty: perioperative factors associated with same-day and early discharge
Source: Arthroplasty. 2026 Oct 12;8:76. doi: 10.1186/s42836-026-00435-1 (PMC13656685; doi:10.1186/s42836-026-00435-1)
Supplement: Supplementary file 1 [file 42836_2026_435_MOESM2_ESM.pdf]

Supplementary Material

Supplementary Table A. Missing data and imputation strategy for model predictors

Variable	Not applicable (structural)	True missing n (%)	Imputation method
Age	–	0 (0.0%)	–
Sex	–	0 (0.0%)	–
Side	–	0 (0.0%)	–
Joint type	–	1 (0.03%)	Mode
ASA grade	–	180 (5.95%)	Mode
Attended Joint School	–	0 (0.0%)	–
Pre-op nutrition given	–	0 (0.0%)	–
Pre-op nutrition volume	685 (not given)	2 (0.07%)	MICE
Spinal only	–	0 (0.0%)	–
Spinal type	201 (no spinal)	4 (0.13%)	Mode
Spinal concentration	201 (no spinal)	4 (0.13%)	MICE
Spinal volume (mL)	201 (no spinal)	68 (2.25%)	MICE
Spinal preparation	201 (no spinal)	4 (0.13%)	Mode
General anaesthesia	–	0 (0.0%)	–
Dexamethasone given	–	0 (0.0%)	–
Dexamethasone dose	237 (not given)	4 (0.13%)	MICE
Nerve block given	–	19 (0.63%)	Mode
Number of nerve blocks	–	0 (0.0%)	–
ACB / FIB / PENG / AFCN	–	0 (0.0%)	–
Anaesthetic protocol followed	–	62 (2.05%)	Mode
Post-op nutrition given	–	0 (0.0%)	–
Post-op nutrition volume	1,505 (not given)	0 (0.0%)	MICE
Day 0 pain	–	30 (0.99%)	Mode
Day 0 mobilisation	–	71 (2.35%)	Mode
Time to mobilisation	–	71 (2.35%)	MICE
First mobiliser designation	–	87 (2.88%)	Mode
Blood transfusion	–	91 (3.01%)	Mode
X-ray on return to ward	–	0 (0.0%)	–

N = 3,025. "Not applicable (structural)" denotes values that cannot exist for that patient — for example spinal drug variables in patients who did not receive a spinal anaesthetic, or nutritional volume in patients not given supplementation. These were coded as zero, representing the quantity actually administered, and were not imputed. The categorical spinal variables retain an explicit "not given" level. "True missing" denotes values that were not recorded but could have existed, and these alone were imputed. Continuous variables were imputed using Multivariate Imputation by Chained Equations (MICE; IterativeImputer) and categorical variables using the most frequent category (mode). All imputation was fitted within the training partition only and applied to held-out data, so that no test-set information contributed to the imputation model. The maximum true missingness for any predictor was 5.95% (ASA grade).

Supplementary Material B. Analysis code

The complete code used for data preparation, correction of data-entry errors, coding of structural non-applicability, imputation, model development, internal and temporal validation, the sensitivity analysis, joint-specific subgroup models, and SHAP figure generation is reproduced below. All random seeds are fixed (seed = 42). Imputation is fitted within the training partition only, SMOTE is applied to the training data only, temporal validation splits on the calendar year of surgery, and SHAP values are computed on the held-out test partition.

```
"""
============================================================================
Supplementary Material B - Analysis code
"Using Machine Learning to Evaluate a Non-Selective Enhanced Recovery Pathway
 for Hip and Knee Arthroplasty: Perioperative Factors Associated With
 Same-Day and Early Discharge"
============================================================================
Reproducibility notes
---------------------
* All random seeds fixed (SEED = 42), including the SVM.
* Values that are STRUCTURALLY NOT APPLICABLE (e.g. spinal drug variables in
  patients who received no spinal) are coded as ZERO - the quantity actually
  administered - and are NOT imputed. Categorical spinal variables retain an
  explicit "not given" level.
* Only genuinely missing values are imputed. Imputation is fitted WITHIN the
  training partition and applied to held-out data (no leakage).
* SMOTE is applied to the TRAINING data only.
* Temporal validation splits on calendar year of surgery (DoS), not row order.
* Positive class = discharge FAILURE for both outcomes.
* Spinal agent and spinal volume are modelled separately. The derived variable
  spinal dose (concentration x volume) was NOT used as a predictor: because
  bupivacaine spans 2.5-33 mg and prilocaine 34-110 mg with no overlap, it acts
  as a perfect proxy for agent and cannot be interpreted as a dose-response.
* Reported: ROC-AUC, PR-AUC, precision, recall, F1, Brier, confusion matrices,
  with baseline prevalence of the positive class.
Software: Python 3.12.3, scikit-learn 1.8.0, imbalanced-learn 0.14.2, shap 0.52.0
============================================================================
"""
import warnings; warnings.filterwarnings("ignore")
import numpy as np, pandas as pd
from collections import Counter
from sklearn.experimental import enable_iterative_imputer         # noqa: F401
from sklearn.impute import IterativeImputer, SimpleImputer
from sklearn.model_selection import train_test_split
from sklearn.compose import ColumnTransformer
from sklearn.preprocessing import OneHotEncoder
from sklearn.pipeline import Pipeline
from sklearn.linear_model import LogisticRegression
from sklearn.svm import SVC
from sklearn.ensemble import RandomForestClassifier, GradientBoostingClassifier
from sklearn.tree import DecisionTreeClassifier
from sklearn.metrics import (roc_auc_score, average_precision_score,
    precision_score, recall_score, f1_score, brier_score_loss, confusion_matrix)
from imblearn.over_sampling import SMOTE

SEED = 42
DATA_PATH = "ERAS_Cleaned_and_coded.xlsx"

PREDICTORS = ["Age","Sex","Side","Joint_","ASA","Spinal type","Spinal Vol (ml)",
    "Spinal preparation type","Spinal Conc","Spinal Only","Dose of DXM (mg)","DXM given","D0 Pain","D0 mob",
    "1st Mob who: designation_","Time to mobilisation","Attended JS",
    "Pre-op Nutrition given","Pre-op Nutrition Volume","Post-op Nutrition Given",
    "Post-op nutrition Volume","Number of Nerve Blocks","Blood transfusion",
    "X-ray on ret to ward","Anaes Prot followed","FIB","ACB","PENG","AFCN"]
CONTINUOUS = ["Age","Spinal Vol (ml)","Spinal Conc","Dose of DXM (mg)","Time to mobilisation",
    "Pre-op Nutrition Volume","Post-op nutrition Volume","Number of Nerve Blocks"]
NEAR_OUTCOME = ["Time to mobilisation","X-ray on ret to ward","D0 Pain","D0 mob",
    "Post-op Nutrition Given","Post-op nutrition Volume","1st Mob who: designation_"]



# ---------------------------------------------------------------------------
# 1) Load, apply data corrections, and code structural non-applicability as zero
# ---------------------------------------------------------------------------
def load_data(path=DATA_PATH):
    df = pd.read_excel(path, sheet_name="Data")

    # Prilocaine is stocked only as 2% hyperbaric (Prilotekal). Records coded as
    # 1%, or as a plain preparation, are data-entry errors and are corrected.
    df.loc[(df["Spinal type"] == 1) & (df["Spinal Conc"] == 1.0), "Spinal Conc"] = 2.0
    df.loc[(df["Spinal type"] == 1) & (df["Spinal preparation type"] != 1),
           "Spinal preparation type"] = 1.0

    # Four patients flagged as having no spinal but with a recorded spinal volume
    # are reclassified as having received a spinal.
    m = (df["Spinal Given"] == 0) & (df["Spinal Vol (ml)"] > 0)
    df.loc[m, "Spinal Given"] = 1
    df.loc[m & (df["GA"] == 0), "Spinal Only"] = 1

    # Structural non-applicability -> ZERO (not missing, not imputed)
    no_spinal = df["Spinal Given"] == 0
    df.loc[no_spinal, ["Spinal Vol (ml)", "Spinal Conc"]] = 0.0
    df.loc[df["DXM given"] == 0, "Dose of DXM (mg)"] = 0.0
    df.loc[df["Pre-op Nutrition given"] == 0, "Pre-op Nutrition Volume"] = 0.0
    df.loc[df["Post-op Nutrition Given"] == 0, "Post-op nutrition Volume"] = 0.0

    # Residual -1 within an applicable subset = TRUE missing -> NaN (imputed)
    for c in ["Spinal Vol (ml)", "Spinal Conc", "Spinal type", "Spinal preparation type"]:
        df.loc[(df["Spinal Given"] == 1) & (df[c] == -1), c] = np.nan
    df.loc[(df["DXM given"] == 1) & (df["Dose of DXM (mg)"] == -1), "Dose of DXM (mg)"] = np.nan

    # Categorical spinal variables keep -1 as an explicit "not given" level
    for c in ["Spinal type", "Spinal preparation type"]:
        df.loc[no_spinal, c] = -1

    df["Time to mobilisation"] = df["Time to mobilisation ( Continous Variable )"]
    df["Y_day01_fail"] = (df["Disharge at Day 0 or 1"] == 0).astype(int)
```

```
    df["Y_day0_fail"] = (df["Day case"] == 0).astype(int)

    print("Outcome distributions (FAILURE = 1):")
    print("  Day 0/1 failure:", dict(Counter(df["Y_day01_fail"])))
    print("  Day 0   failure:", dict(Counter(df["Y_day0_fail"])))
    return df


def build_preprocessor(predictors):
    """MICE for continuous, mode + one-hot for categorical. Fitted on train only."""
    cont = [c for c in CONTINUOUS if c in predictors]
    cat = [c for c in predictors if c not in cont]
    return ColumnTransformer([
        ("num", Pipeline([("mice", IterativeImputer(random_state=SEED, max_iter=10))]), cont),
        ("cat", Pipeline([("mode", SimpleImputer(strategy="most_frequent")),
                          ("onehot", OneHotEncoder(handle_unknown="ignore"))]), cat)])


def build_models():
    return {
        "Logistic": LogisticRegression(C=1, max_iter=5000),
        "SVM": SVC(C=10, kernel="linear", probability=True, random_state=SEED),
        "RandomForest": RandomForestClassifier(n_estimators=200, max_depth=10,
                                               random_state=SEED),
        "GradientBoosting": GradientBoostingClassifier(n_estimators=200,
                             learning_rate=0.05, max_depth=3, random_state=SEED),
        "DecisionTree": DecisionTreeClassifier(max_depth=10, min_samples_split=10,
                                               random_state=SEED)}


def run_split(X_train, y_train, X_test, y_test, predictors, title):
    print(f"\n{'=' * 78}\n{title}")
    print(f"Train n = {len(y_train)} | Test n = {len(y_test)} | "
          f"baseline prevalence (failure) = {np.mean(y_test) * 100:.1f}%")
    prep = build_preprocessor(predictors)
    Z_tr = prep.fit_transform(X_train)          # fitted on TRAIN only
    Z_te = prep.transform(X_test)               # test data transformed only
    if hasattr(Z_tr, "todense"):
        Z_tr, Z_te = np.asarray(Z_tr.todense()), np.asarray(Z_te.todense())
    Z_res, y_res = SMOTE(random_state=SEED).fit_resample(Z_tr, y_train)

    rows = []
    for name, model in build_models().items():
        model.fit(Z_res, y_res)
        p = model.predict_proba(Z_te)[:, 1]
        yh = (p >= 0.5).astype(int)
        rows.append({"Model": name,
            "ROC_AUC": round(roc_auc_score(y_test, p), 3),
            "PR_AUC": round(average_precision_score(y_test, p), 3),
            "Precision": round(precision_score(y_test, yh, zero_division=0), 3),
            "Recall": round(recall_score(y_test, yh, zero_division=0), 3),
            "F1": round(f1_score(y_test, yh, zero_division=0), 3),
            "Brier": round(brier_score_loss(y_test, p), 3),
            "ConfusionMatrix": confusion_matrix(y_test, yh).tolist()})
        print(f"  {name:18s} ROC {rows[-1]['ROC_AUC']:.3f} | PR-AUC {rows[-1]['PR_AUC']:.3f} "
              f"| F1 {rows[-1]['F1']:.3f} | CM {rows[-1]['ConfusionMatrix']}")
    return pd.DataFrame(rows)


def main():
    data = load_data()
    predictors = [p for p in PREDICTORS if p in data.columns]
    X_all = data[predictors]

    # Tables 4 and 5 - internal validation (80/20 stratified)
    for ycol, title in [("Y_day01_fail", "TABLE 4 - Day 0/1 discharge failure (80/20)"),
                        ("Y_day0_fail", "TABLE 5 - Day 0 discharge failure (80/20)")]:
        y = data[ycol]
        X_tr, X_te, y_tr, y_te = train_test_split(X_all, y, test_size=0.2,
                                                  stratify=y, random_state=SEED)
        run_split(X_tr, y_tr.values, X_te, y_te.values, predictors, title)

    # Supplementary Tables C and D - temporal validation by calendar year
    year = pd.to_datetime(data["DoS"], dayfirst=True).dt.year
    tr, te = (year == 2024), (year == 2025)
    for ycol, title in [("Y_day01_fail", "SUPP C - Day 0/1 failure (2024 -> 2025)"),
                        ("Y_day0_fail", "SUPP D - Day 0 failure (2024 -> 2025)")]:
        y = data[ycol]
        run_split(X_all[tr], y[tr].values, X_all[te], y[te].values, predictors, title)

    # Supplementary Table H - sensitivity analysis (no near-outcome variables)
    reduced = [p for p in predictors if p not in NEAR_OUTCOME]
    for ycol, lab in [("Y_day0_fail", "DAY 0"), ("Y_day01_fail", "DAY 0/1")]:
        y = data[ycol]
        X_tr, X_te, y_tr, y_te = train_test_split(data[reduced], y, test_size=0.2,
                                                  stratify=y, random_state=SEED)
        run_split(X_tr, y_tr.values, X_te, y_te.values, reduced,
                  f"SUPP H - {lab}: preoperative + intraoperative variables only")

    # Supplementary Table L - subgroup models by joint type
    for j, jn in [(0, "THR"), (1, "TKR")]:
        sub = data[data["Joint_"] == j]
        P = [p for p in predictors if p != "Joint_" and sub[p].nunique(dropna=True) > 1]
        P = [p for p in P if not (p in ["ACB", "FTB", "PENG", "AFCN"] and (sub[p] == 1).sum() < 20)]
        for ycol, lab in [("Y_day0_fail", "Day 0"), ("Y_day01_fail", "Day 0/1")]:
            y = sub[ycol]
            X_tr, X_te, y_tr, y_te = train_test_split(sub[P], y, test_size=0.2,
                                                      stratify=y, random_state=SEED)
            run_split(X_tr, y_tr.values, X_te, y_te.values, P,
                      f"SUPP L - {jn}: {lab} failure")
    # Partial knee replacement is not modelled separately: 123 patients with only
    # six Day 0/1 discharge failures gives 0.2 events per candidate predictor.


# ---------------------------------------------------------------------------
# SHAP (Figures 3-4, Supplementary Tables F, G and J) - test partition only
# ---------------------------------------------------------------------------
```

```
def shap_analysis():
    import shap, matplotlib
    matplotlib.use("Agg")
    import matplotlib.pyplot as plt
    data = load_data()
    predictors = [p for p in PREDICTORS if p in data.columns]
    X_all = data[predictors]
    cont = [c for c in CONTINUOUS if c in predictors]
    cat = [c for c in predictors if c not in cont]

    for ycol, title, fname in [
            ("Y_day0_fail", "SHAP: Day 0 (same-day) discharge failure", "figure4_shap_day0"),
            ("Y_day01_fail", "SHAP: Day 0 or Day 1 discharge failure", "figure3_shap_day01")]:
        y = data[ycol]
        X_tr, X_te, y_tr, y_te = train_test_split(X_all, y, test_size=0.2,
                                                   stratify=y, random_state=SEED)
        X_tr2, X_te2 = X_tr.copy(), X_te.copy()
        mice = IterativeImputer(random_state=SEED, max_iter=10)
        X_tr2[cont] = mice.fit_transform(X_tr[cont])       # fitted on TRAIN only
        X_te2[cont] = mice.transform(X_te[cont])
        mode = SimpleImputer(strategy="most_frequent")
        X_tr2[cat] = mode.fit_transform(X_tr[cat])
        X_te2[cat] = mode.transform(X_te[cat])
        Z_res, y_res = SMOTE(random_state=SEED).fit_resample(X_tr2.values, y_tr.values)
        model = GradientBoostingClassifier(n_estimators=200, learning_rate=0.05,
                                           max_depth=3, random_state=SEED).fit(Z_res, y_res)
        # SHAP computed on the HELD-OUT TEST PARTITION only
        shap_values = shap.TreeExplainer(model).shap_values(X_te2.values)
        pd.Series(np.abs(shap_values).mean(0), index=predictors).sort_values(
            ascending=False).round(4).to_csv(f"{fname}_values.csv", header=["Mean_abs_SHAP"])
        plt.figure()
        shap.summary_plot(shap_values, X_te2.values, feature_names=predictors,
                          max_display=15, show=False, plot_size=(9, 7))
        plt.title(title, fontsize=13, fontweight="bold", pad=12)
        plt.xlabel("SHAP value (impact on predicted discharge failure)", fontsize=10)
        plt.tight_layout()
        plt.savefig(f"{fname}.tiff", dpi=600, bbox_inches="tight", format="tiff")
        plt.close()
        print(f"Saved {fname}.tiff and {fname}_values.csv")


if __name__ == "__main__":
    main()
    shap_analysis()
```

Supplementary Table C. Temporal validation — failure of Day 0 or Day 1 discharge (2024 → 2025)

Model	ROC-AUC	PR-AUC	Precision	Recall	F1	Brier
Logistic Regression	0.761	0.481	0.366	0.549	0.439	0.176
Support Vector Machine	0.759	0.483	0.375	0.534	0.441	0.174
Random Forest	0.716	0.382	0.561	0.180	0.272	0.135
Gradient Boosting	0.749	0.437	0.612	0.252	0.357	0.129
Decision Tree	0.611	0.272	0.331	0.252	0.287	0.185

Training set: patients treated in 2024 (n = 1,898). Test set: patients treated in 2025 (n = 1,127). The split is performed on the calendar year of surgery. Positive class = failure of Day 0 or Day 1 discharge; baseline prevalence in the test set = 18.3%, so a no-skill classifier would achieve a PR-AUC of approximately 0.18.

Supplementary Table D. Temporal validation — failure of Day 0 (same-day) discharge (2024 → 2025)

Model	ROC-AUC	PR-AUC	Precision	Recall	F1	Brier
Logistic Regression	0.810	0.932	0.908	0.668	0.770	0.183
Support Vector Machine	0.809	0.932	0.913	0.661	0.767	0.182
Random Forest	0.803	0.929	0.843	0.787	0.814	0.157
Gradient Boosting	0.811	0.932	0.845	0.803	0.823	0.154
Decision Tree	0.751	0.885	0.854	0.748	0.798	0.203

Training set: patients treated in 2024 (n = 1,898). Test set: patients treated in 2025 (n = 1,127). Positive class = failure of Day 0 discharge; baseline prevalence in the test set = 73.7%, so a no-skill classifier would achieve a PR-AUC of approximately 0.74. The high F1 and PR-AUC values therefore partly reflect majority-class prevalence and should be interpreted against this baseline.

Supplementary Material E. Calibration curves and confusion matrices

Calibration curves for all models on the held-out test partition are provided as a separate image file (Supplementary Figure E). Predicted probabilities are grouped into ten quantile bins and plotted against observed event frequency. The diagonal represents perfect calibration. Gradient Boosting and Random Forest were the best calibrated (Brier 0.132–0.155), whereas Logistic Regression was least well calibrated despite high discrimination (Brier 0.181–0.200).

Model	Day 0 confusion matrix	Day 0 or Day 1 confusion matrix
Logistic Regression	TN 133, FP 29, FN 149, TP 294	TN 359, FP 137, FN 37, TP 72
Random Forest	TN 93, FP 69, FN 81, TP 362	TN 468, FP 28, FN 79, TP 30
Gradient Boosting	TN 91, FP 71, FN 79, TP 364	TN 465, FP 31, FN 74, TP 35
Decision Tree	TN 84, FP 78, FN 92, TP 351	TN 450, FP 46, FN 70, TP 39

Confusion matrices on the held-out test partition (n = 605) at a classification threshold of 0.5. Positive class = discharge failure.

Supplementary Table F. SHAP feature importance — Day 0 or Day 1 discharge failure

Mean absolute SHAP values for all 29 predictors, computed from the Gradient Boosting model on the held-out test partition, are provided as a separate file (figure3_shap_day01_values.csv). The eight highest-ranked features are shown below.

Feature	Mean |SHAP|
ASA grade	0.4089
Sex	0.3269
Operative side	0.3165
Time to mobilisation	0.3094
Number of nerve blocks	0.2987
X-ray on return to ward	0.2600
Age	0.2077
Adductor canal block	0.1907

Supplementary Table G. SHAP feature importance — Day 0 (same-day) discharge failure

Mean absolute SHAP values for all 29 predictors, computed from the Gradient Boosting model on the held-out test partition, are provided as a separate file (figure4_shap_day0_values.csv). The eight highest-ranked features are shown below.

Feature	Mean |SHAP|
Time to mobilisation	1.2115
X-ray on return to ward	0.3000
Mobiliser designation	0.2552
Sex	0.1900
Operative side	0.1899
Age	0.1838
Spinal volume (mL)	0.1532
Spinal agent	0.1408

Supplementary Table H. Sensitivity analysis — preoperative and intraoperative variables only

Outcome	Model	Full ROC-AUC	Reduced ROC-AUC	Δ ROC	Full PR-AUC	Reduced PR-AUC
Day 0	Logistic Regression	0.809	0.631	−0.177	0.930	0.825
Day 0	Support Vector Machine	0.806	0.631	−0.176	0.929	0.825
Day 0	Random Forest	0.811	0.643	−0.168	0.931	0.821
Day 0	Gradient Boosting	0.815	0.642	−0.173	0.931	0.828
Day 0	Decision Tree	0.761	0.633	−0.128	0.886	0.808
Day 0/1	Logistic Regression	0.760	0.722	−0.039	0.497	0.430
Day 0/1	Support Vector Machine	0.757	0.720	−0.037	0.493	0.429
Day 0/1	Random Forest	0.728	0.680	−0.048	0.390	0.348
Day 0/1	Gradient Boosting	0.744	0.707	−0.037	0.436	0.421
Day 0/1	Decision Tree	0.663	0.611	−0.052	0.336	0.259

The reduced model excludes seven near-outcome variables measured during or concurrent with the discharge process: time to mobilisation, X-ray on return to ward, Day 0 pain, Day 0 mobilisation, post-operative nutrition given, post-operative nutrition volume, and first-mobiliser designation. 80/20 stratified split; test n = 605. Removing these variables reduced discrimination for same-day discharge substantially (ROC-AUC ≈ 0.81 to ≈ 0.64), whereas Day 0 or Day 1 discharge was comparatively preserved (≈ 0.76 to ≈ 0.73). This indicates that same-day discharge is predicted largely by intra-process events rather than by upstream, preoperatively modifiable factors, and supports the framing of these variables as associations requiring prospective confirmation rather than as causal determinants.

Supplementary Table I. Model hyperparameters and implementation

Model	Hyperparameters specified
Logistic Regression	C = 1; max_iter = 5000
Support Vector Machine	C = 10; kernel = linear; probability = True; random_state = 42
Random Forest	n_estimators = 200; max_depth = 10; random_state = 42
Gradient Boosting	n_estimators = 200; learning_rate = 0.05; max_depth = 3; random_state = 42
Decision Tree	max_depth = 10; min_samples_split = 10; random_state = 42
**Implementation step**	**Setting**
Internal validation	80/20 stratified split; random_state = 42
Temporal validation	Split on calendar year of surgery (2024 train, n = 1,898; 2025 test, n = 1,127)
Class imbalance	SMOTE (random_state = 42), training partition only
Imputation	MICE (continuous) / mode (categorical), fitted on training partition only
Structural non-applicability	Coded as zero; not imputed
Classification threshold	0.5
Software	Python 3.12.3; scikit-learn 1.8.0; imbalanced-learn 0.14.2; shap 0.52.0

Scikit-learn defaults were retained for all hyperparameters not listed. Hyperparameters were specified a priori on the basis of prior optimisation and clinical interpretability rather than iterative grid searching.

Supplementary Table J. Cross-model comparison of SHAP feature rankings

Day 0 (same-day) discharge failure — rank by mean |SHAP| within each model:

Feature	Gradient Boosting	Random Forest	Logistic Regression
Time to mobilisation	1	1	1
X-ray on return to ward	2	3	4
Mobiliser designation	3	4	3
Sex	4	5	7
Operative side	5	15	20
Age	6	8	5
Spinal volume	7	9	22
Spinal agent	8	6	9

Day 0 or Day 1 discharge failure — rank by mean |SHAP| within each model:

Feature	Gradient Boosting	Random Forest	Logistic Regression
ASA grade	1	1	6
Sex	2	2	5
Operative side	3	14	14
Time to mobilisation	4	4	1
Number of nerve blocks	5	9	12
X-ray on return to ward	6	3	7
Age	7	5	10
Adductor canal block	8	10	21

Features are ordered by their Gradient Boosting rank. Time to mobilisation ranked first for same-day discharge in all three models, indicating that the dominant finding is robust to model choice. Agreement was weaker among lower-ranked features: only three of the top five features for same-day discharge, and two of the top five for Day 0 or Day 1 discharge, were common to all three models. Lower-ranked associations should therefore be interpreted with caution.

Supplementary Table K. Comparison of practice and outcomes between 2024 and 2025

Variable	2024 (n = 1,898)	2025 (n = 1,127)	p
Day 0 discharge	27.1%	26.3%	0.602
Day 0 or Day 1 discharge	82.2%	81.7%	0.745
Anaesthetic protocol adherence	90.2%	94.4%	< 0.001
Joint School attendance	83.3%	87.5%	0.002
Spinal-only anaesthesia	88.3%	90.7%	0.042
Pre-op nutrition given	76.0%	79.6%	0.024
X-ray on return to ward	79.8%	83.0%	0.033
Dexamethasone given	92.9%	90.9%	0.040
Mean spinal volume (mL) †	3.24	3.38	< 0.001
Median time to mobilisation (h)	2.83	3.00	0.753
ASA grade 3 or 4	18.2%	17.2%	–
Median age (years)	69.9	70.4	–

Categorical comparisons used the chi-square test without continuity correction; continuous comparisons used the Mann–Whitney U test. Percentages are calculated on non-missing denominators. † Among spinal recipients. Discharge outcomes and case mix were stable between periods, whereas several process measures shifted significantly, all in the direction of increasing protocol compliance. The pathway therefore matured over the study period while its outputs remained stable.

Supplementary Table L. Joint-specific subgroup models

Joint	Outcome	Model	ROC-AUC	PR-AUC	F1	Baseline
THA	Day 0	Logistic Regression	0.883	0.959	0.801	74.0%
THA	Day 0	Support Vector Machine	0.884	0.960	0.787	
THA	Day 0	Random Forest	0.878	0.958	0.856	
THA	Day 0	Gradient Boosting	0.864	0.954	0.859	
THA	Day 0	Decision Tree	0.797	0.895	0.864	
THA	Day 0/1	Logistic Regression	0.833	0.555	0.552	17.8%
THA	Day 0/1	Support Vector Machine	0.828	0.540	0.561	
THA	Day 0/1	Random Forest	0.773	0.454	0.371	
THA	Day 0/1	Gradient Boosting	0.791	0.502	0.447	
THA	Day 0/1	Decision Tree	0.700	0.375	0.382	
TKA	Day 0	Logistic Regression	0.814	0.938	0.757	74.7%
TKA	Day 0	Support Vector Machine	0.811	0.936	0.740	
TKA	Day 0	Random Forest	0.837	0.945	0.845	
TKA	Day 0	Gradient Boosting	0.828	0.944	0.838	
TKA	Day 0	Decision Tree	0.708	0.864	0.788	
TKA	Day 0/1	Logistic Regression	0.730	0.520	0.439	18.9%
TKA	Day 0/1	Support Vector Machine	0.727	0.520	0.425	
TKA	Day 0/1	Random Forest	0.708	0.463	0.370	
TKA	Day 0/1	Gradient Boosting	0.698	0.508	0.409	
TKA	Day 0/1	Decision Tree	0.685	0.362	0.370	

Models were refitted separately within each joint subgroup, excluding joint type as a predictor and excluding block types administered to fewer than 20 patients within the subgroup. Baseline denotes the prevalence of the positive class (discharge failure) in the test partition. Partial knee replacement was not modelled separately: with 123 patients and only six Day 0 or Day 1 discharge failures, the number of events per candidate predictor was 0.2, far below that required for stable estimation. Hip-specific models discriminated better than the pooled models for both outcomes.

Supplementary Table M. Univariate effect sizes for all candidate factors

Odds ratios are for achieving the stated discharge outcome. For continuous variables the effect is the difference in means between patients achieving and not achieving discharge, with the 95% confidence interval.

Failure of Day 0 or Day 1 discharge — outcome achieved:

Comparison	Exposed n (%)	Reference n (%)	OR or mean difference (95% CI)	p
Male vs. female	1301/1519 (85.6%)	1180/1506 (78.4%)	1.65 (1.37–1.99)	< 0.001
ASA 1 vs. ASA 2	198/213 (93.0%)	1807/2092 (86.4%)	2.08 (1.21–3.57)	0.007
ASA 3 vs. ASA 2	338/537 (62.9%)	1807/2092 (86.4%)	0.27 (0.22–0.33)	< 0.001
Bilateral vs. unilateral	10/33 (30.3%)	2471/2992 (82.6%)	0.09 (0.04–0.19)	< 0.001
PKR vs. THA	117/123 (95.1%)	1101/1343 (82.0%)	4.29 (1.86–9.85) †	< 0.001
TKA vs. THA	1262/1558 (81.0%)	1101/1343 (82.0%)	0.94 (0.78–1.13)	0.499
Joint School attended vs. not	2125/2567 (82.8%)	356/458 (77.7%)	1.38 (1.08–1.76)	0.010
Pre-op nutrition vs. none	1955/2340 (83.5%)	526/685 (76.8%)	1.53 (1.25–1.89)	< 0.001
Spinal + GA vs. spinal alone	94/126 (74.6%)	2246/2698 (83.2%)	0.59 (0.39–0.89)	0.012
GA alone vs. spinal alone	131/185 (70.8%)	2246/2698 (83.2%)	0.49 (0.35–0.68)	< 0.001
Prilocaine vs. bupivacaine	562/626 (89.8%)	1775/2194 (80.9%)	2.07 (1.57–2.74)	< 0.001
Bupivacaine 0.5% vs. 0.25%	978/1237 (79.1%)	767/921 (83.3%)	0.76 (0.61–0.95)	0.014
Heavy vs. plain (bupivacaine)	490/631 (77.7%)	1285/1563 (82.2%)	0.75 (0.60–0.94)	0.014
Dexamethasone vs. none	2300/2788 (82.5%)	181/237 (76.4%)	1.46 (1.06–2.00)	0.018
Protocol followed vs. not	2263/2719 (83.2%)	164/244 (67.2%)	2.42 (1.82–3.22)	< 0.001
Nerve block vs. none	1371/1682 (81.5%)	1095/1324 (82.7%)	0.92 (0.76–1.11)	0.397
ACB vs. none (knee)	1181/1435 (82.3%)	81/123 (65.9%)	2.41 (1.62–3.58)	< 0.001
FIB vs. none (knee)	49/81 (60.5%)	1213/1477 (82.1%)	0.33 (0.21–0.53)	< 0.001
PENG vs. none (hip)	41/55 (74.5%)	1060/1288 (82.3%)	0.63 (0.34–1.17)	0.143
X-ray on return vs. none	2115/2450 (86.3%)	366/575 (63.7%)	3.61 (2.94–4.43)	< 0.001
Physiotherapy/ERP vs. nurse	2074/2498 (83.0%)	343/440 (78.0%)	1.38 (1.08–1.77)	0.010
Post-op nutrition vs. none	1331/1520 (87.6%)	1150/1505 (76.4%)	2.17 (1.79–2.64)	< 0.001
Transfusion vs. none	8/24 (33.3%)	2433/2910 (83.6%)	0.10 (0.04–0.23) †	< 0.001
Moderate/severe vs. no Day 0 pain	150/203 (73.9%)	1818/2168 (83.9%)	0.54 (0.39–0.76)	< 0.001
Age, years (mean)	68.25	72.15	-3.89 (-4.90 to -2.89)	< 0.001
Spinal volume, bupivacaine (mL)	3.26	3.16	+0.10 (+0.01 to +0.18)	0.002
Time to mobilisation (hours)	4.81	9.28	-4.48 (-5.35 to -3.61)	< 0.001

Same-day (Day 0) discharge — outcome achieved:

Comparison	Exposed n (%)	Reference n (%)	OR or mean difference (95% CI)	p
Male vs. female	472/1519 (31.1%)	339/1506 (22.5%)	1.55 (1.32–1.83)	< 0.001
ASA 1 vs. ASA 2	75/213 (35.2%)	612/2092 (29.3%)	1.31 (0.98–1.77)	0.070
ASA 3 vs. ASA 2	83/537 (15.5%)	612/2092 (29.3%)	0.44 (0.34–0.57)	< 0.001
Bilateral vs. unilateral	4/33 (12.1%)	807/2992 (27.0%)	0.37 (0.13–1.07) †	0.073
PKR vs. THA	67/123 (54.5%)	349/1343 (26.0%)	3.41 (2.34–4.96)	< 0.001
TKA vs. THA	394/1558 (25.3%)	349/1343 (26.0%)	0.96 (0.82–1.14)	0.668
Joint School attended vs. not	706/2567 (27.5%)	105/458 (22.9%)	1.28 (1.01–1.61)	0.042
Pre-op nutrition vs. none	642/2340 (27.4%)	169/685 (24.7%)	1.15 (0.95–1.40)	0.151
Spinal + GA vs. spinal alone	28/126 (22.2%)	747/2698 (27.7%)	0.75 (0.49–1.15)	0.179
GA alone vs. spinal alone	33/185 (17.8%)	747/2698 (27.7%)	0.57 (0.39–0.83)	0.004
Prilocaine vs. bupivacaine	268/626 (42.8%)	505/2194 (23.0%)	2.50 (2.08–3.02)	< 0.001
Bupivacaine 0.5% vs. 0.25%	260/1237 (21.0%)	241/921 (26.2%)	0.75 (0.61–0.92)	0.005
Heavy vs. plain (bupivacaine)	132/631 (20.9%)	373/1563 (23.9%)	0.84 (0.67–1.06)	0.138
Dexamethasone vs. none	761/2788 (27.3%)	50/237 (21.1%)	1.40 (1.02–1.94)	0.039
Protocol followed vs. not	755/2719 (27.8%)	41/244 (16.8%)	1.90 (1.35–2.69)	< 0.001
Nerve block vs. none	458/1682 (27.2%)	347/1324 (26.2%)	1.05 (0.90–1.24)	0.530

ACB vs. none (knee)	373/1435 (26.0%)	21/123 (17.1%)	1.71 (1.05–2.77)	0.029
FIB vs. none (knee)	11/81 (13.6%)	383/1477 (25.9%)	0.45 (0.24–0.86)	0.013
PENG vs. none (hip)	11/55 (20.0%)	338/1288 (26.2%)	0.70 (0.36–1.38)	0.301
X-ray on return vs. none	794/2450 (32.4%)	17/575 (3.0%)	15.74 (9.64–25.69)	< 0.001
Physiotherapy/ERP vs. nurse	796/2498 (31.9%)	11/440 (2.5%)	18.24 (9.97–33.38)	< 0.001
Post-op nutrition vs. none	496/1520 (32.6%)	315/1505 (20.9%)	1.83 (1.55–2.16)	< 0.001
Transfusion vs. none	2/24 (8.3%)	801/2910 (27.5%)	0.24 (0.06–1.02) †	0.037
Moderate/severe vs. no Day 0 pain	42/203 (20.7%)	598/2168 (27.6%)	0.68 (0.48–0.97)	0.034
Age, years (mean)	66.93	69.70	-2.76 (-3.63 to -1.89)	< 0.001
Spinal volume, bupivacaine (mL)	3.30	3.23	+0.07 (+0.00 to +0.14)	0.020
Time to mobilisation (hours)	2.01	6.95	-4.94 (-5.25 to -4.62)	< 0.001

Odds ratios greater than 1 indicate higher odds of achieving discharge. Categorical comparisons used the chi-square test without continuity correction, or Fisher's exact test where any cell contained fewer than ten observations; continuous comparisons used the Mann–Whitney U test. † At least one cell contained fewer than ten observations; the Wald confidence interval is unreliable in this setting and the exact p-value should be preferred. ACB = adductor canal block; ERP = enhanced recovery practitioner; FIB = fascia iliaca block; GA = general anaesthesia; PENG = pericapsular nerve group block.

Supplementary Material N. TRIPOD+AI reporting checklist

Section / Topic	Item	Checklist item	Reported in manuscript
Title	1	Identify the study as developing and/or validating a prediction model, specify target population and outcome.	Title page, line 2. Identified as an evaluation of a perioperative pathway, not a deployable prediction model.
Abstract	2	Provide a structured summary of objectives, design, setting, participants, sample size, predictors, outcome, statistical analysis, results and conclusions.	Abstract, lines 36–65.
Introduction	3a	Explain the medical context and rationale for developing or validating the model.	Introduction, lines 69–78.
Introduction	3b	Specify the objectives, including whether the study describes development, validation, or both.	Introduction, lines 79–88. Stated as pathway evaluation rather than preoperative prediction.
Methods — Source of data	4a	Describe the study design or source of data.	Methods, line 105. Prospectively collected clinical data from a single tertiary centre.
Methods — Source of data	4b	Specify the key study dates.	Methods, line 94. April 2024 to May 2025.
Methods — Participants	5a	Specify key elements of the study setting.	Methods, lines 102–104. Universal non-selective ERAS pathway; components in Figure 1, anaesthetic protocol in Figure 2.
Methods — Participants	5b	Describe eligibility criteria for participants.	Methods, lines 95–101. All primary THA, TKA and PKR; no exclusion by age, comorbidity or anticipated day-case suitability.
Methods — Participants	5c	Give details of treatments received, if relevant.	Methods, lines 102–104 and Tables 2–3.
Methods — Outcome	6a	Clearly define the outcome that is predicted, including how and when assessed.	Methods, lines 120–121. Failure of Day 0 or Day 1 discharge, and failure of same-day (Day 0) discharge.
Methods — Outcome	6b	Report any actions to blind assessment of the outcome.	Not applicable. Discharge date is an administrative record, assessed independently of predictors.
Methods — Predictors	7a	Clearly define all predictors, including how and when they were measured.	Methods, lines 142–149 and Supplementary Table A. Time to mobilisation defined at line 116.
Methods — Predictors	7b	Report any actions to blind assessment of predictors.	Not applicable. Predictors were recorded prospectively during routine care before the outcome was known.
Methods — Sample size	8	Explain how the study size was arrived at.	Methods, lines 95–101 (consecutive sample, no a priori size calculation). Event counts in Results, lines 50–56.
Methods — Missing data	9	Describe how missing data were handled, with details of any imputation method.	Methods, lines 107–115 and Supplementary Table A. Structural non-applicability coded as zero; true missing values imputed within the training partition only.
Methods — Statistical analysis	10a	Describe how predictors were handled in the analysis.	Methods, lines 107–118 and 142–149.
Methods — Statistical analysis	10b	Specify the type of model, all model-building procedures, and method for internal validation.	Methods, lines 119–129. Five algorithms; SMOTE on training data only; 80/20 stratified split; hyperparameters in Supplementary Table I.

Section / Topic	Item	Checklist item	Reported in manuscript
Methods — Statistical analysis	10c	For validation, describe how the predictions were calculated.	Methods, lines 122–139. Internal 80/20 split and temporal split by calendar year (2024 training, n = 1,898; 2025 test, n = 1,127).
Methods — Statistical analysis	10d	Specify all measures used to assess model performance.	Methods, line 140. ROC-AUC, PR-AUC, precision, recall, F1, Brier score and confusion matrices.
Methods — Statistical analysis	10e	Describe any model updating arising from the validation.	Not applicable. No model updating was performed between validation stages.
Methods — Risk groups	11	Provide details on how risk groups were created, if done.	Not applicable. No risk stratification was undertaken; the models were used for pathway evaluation.
Methods — Development vs. validation	12	For validation, identify any differences from the development data.	Methods, lines 137–139. Temporal validation used the same centre and pathway in a later calendar year.
AI-specific — Data preprocessing	AI-1	Describe data preprocessing, including handling of structurally non-applicable values and class imbalance.	Methods, lines 107–118 and 123. SMOTE applied to the training partition only.
AI-specific — Reproducibility	AI-2	Report software, package versions and random seeds sufficient for reproduction.	Methods, lines 127–131. Python 3.12.3, scikit-learn 1.8.0, imbalanced-learn 0.14.2, shap 0.52.0; random_state = 42; classification threshold 0.5.
AI-specific — Leakage	AI-3	State measures taken to prevent information leakage between training and test data.	Methods, lines 128–129. Imputation, resampling and model fitting confined to the training partition; SHAP computed on the held-out test partition only.
AI-specific — Interpretability	AI-4	Describe how model outputs were interpreted and the limitations of that interpretation.	Methods, lines 150–151; Results, lines 272–283; Limitations, lines 368–372. SHAP with cross-model comparison in Supplementary Table J.
Results — Participants	13a	Describe the flow of participants, including the number with and without the outcome.	Results, lines 50–56. 3,025 patients; Day 0 discharge 811 (26.8%); Day 0 or Day 1 discharge 2,481 (82.0%).
Results — Participants	13b	Describe the characteristics of the participants, including the number with missing data.	Results, lines 50–56; Tables 1–3; Supplementary Table A.
Results — Participants	13c	For validation, show a comparison with the development data.	Discussion, lines 316–322 and Supplementary Table K. Case mix and outcomes stable between 2024 and 2025; four process measures shifted.
Results — Model development	14a	Specify the number of participants and outcome events in each analysis.	Results, lines 255–271; Tables 4–5; Supplementary Tables C–D. Baseline failure prevalence reported for each outcome.
Results — Model development	14b	If done, report the unadjusted association between each candidate predictor and outcome.	Results, lines 160–254; Supplementary Table M (odds ratios with 95% confidence intervals for all univariate comparisons).
Results — Model performance	15	Report performance measures for the prediction model.	Results, lines 255–271; Tables 4–5; Supplementary Tables C–D; calibration curves and confusion matrices in Supplementary Material E.

Section / Topic	Item	Checklist item	Reported in manuscript
Results — Model presentation	16	Present the full prediction model or provide access to the code.	Supplementary Material B (complete analysis code); Methods, line 152.
Discussion — Limitations	18	Discuss any limitations, including non-representative sample and missing data.	Discussion, lines 362–380. Four limitations: process-embedded variables, model-dependent SHAP rankings, unrecorded complications, single centre.
Discussion — Interpretation	19a	For validation, discuss the results with reference to performance in the development data.	Discussion, lines 305–322. Train-to-test and temporal comparisons reported.
Discussion — Interpretation	19b	Give an overall interpretation of the results, considering objectives, limitations and other relevant evidence.	Discussion, lines 346–361; Conclusion.
Discussion — Implications	20	Discuss the potential clinical use of the model and implications for future research.	Discussion, lines 336–345 and 352–361. Framed as hypothesis-generating; prospective evaluation required.
Other — Supplementary	21	Provide information about the availability of supplementary resources.	Supplementary Materials A–N, cited throughout Methods and Results.
Other — Funding	22	Give the source of funding and the role of the funders.	Declarations.

Abbreviations: ERAS, enhanced recovery after surgery; PKR, partial knee replacement; PR-AUC, precision–recall area under the curve; ROC-AUC, receiver operating characteristic area under the curve; SHAP, SHapley Additive exPlanations; SMOTE, synthetic minority over-sampling technique; THA, total hip arthroplasty; TKA, total knee arthroplasty.
